# Causal Relationship Between Social Isolation, Loneliness, and Cardiovascular Disease

**DOI:** 10.1192/j.eurpsy.2025.395

**Published:** 2025-08-26

**Authors:** Y. Nie, Y. He, J. Zhang, Y. Y. Liang

**Affiliations:** 1 Center for Sleep and Circadian Medicine, The Affiliated Brain Hospital of Guangzhou Medical University; 2 Department of Medicine, The Third Affiliated Hospital of Sun Yat-Sen University, Guangzhou, China

## Abstract

**Introduction:**

Cardiovascular disease (CVD) has become one of the leading causes of death worldwide. Psychosocial factors play a significant role in the pathogenesis of CVD. Social isolation and loneliness, two important aspects of social determinants, have been shown to be associated with adverse health outcomes. However, it remains unclear whether social isolation and loneliness increase the risk of CVD and exacerbate CVD development.

**Objectives:**

This study aims to investigate 1) whether social isolation and loneliness are associated with CVD; 2) whether the associations between social isolation and loneliness and CVD are consistent with causal effects; and 3) whether social isolation exacerbates CVD development.

**Methods:**

We capitalized on a large sample of individual-level data from the UK Biobank. Participants’ levels of social isolation and loneliness were assessed via questionnaires. 6 cardiovascular diseases (atrial fibrillation, heart failure, hypertension, myocardial infarction, peripheral vascular disease, and stroke) were included by linking hospital admission data and death registry records. We used Cox proportional hazards models to estimate the hazard ratios (HRs) of social isolation and loneliness with the risk of CVD. Additionally, we employed two-sample Mendelian randomization analysis to estimate the causal relationships between social isolation, loneliness, and CVD. Finally, we evaluated the impact of social isolation on the clinical outcomes of myocardial infarction in mice. We determined infarct size using histology and heart function using echocardiography.

**Results:**

During a median follow-up of 12.6 years, after adjusting for demographic, socioeconomic, and lifestyle factors, most isolated group was associated with an increased risk of 4 out of 6 cardiovascular diseases compared to the least isolated group: heart failure, peripheral vascular disease, stroke, myocardial infarction, and hypertension. Loneliness increased the risk of all 6 cardiovascular diseases. Two-sample Mendelian randomization analysis showed that fewer leisure or social activities was associated with an increased risk of stroke (OR 1.01; 95% CI 1.00-1.02). Loneliness was associated with an increased risk of heart failure (OR 1.70; 95% CI 1.13-2.57), myocardial infarction (OR 1.02; 95% CI 1.00-1.04), and atrial fibrillation (OR 1.00; 95% CI 0.99-1.00). Further, compared to group-housed mice, socially isolated mice showed significantly reduced cardiac ejection function, increased myocardial fibrosis and cardiomyocyte apoptosis after myocardial infarction.

**Conclusions:**

Potential causal relationships were identified between genetic liability to social isolation, loneliness and the incidence of cardiovascular diseases. Social isolation may exacerbate the progression of myocardial infarction. The results of this study emphasize the adverse impact of social disconnections on the incidence and development of cardiovascular diseases.

**Disclosure of Interest:**

None Declared

